# Good News: Family Medicine Specialists Have More Opportunity to Diagnose Undiagnosed Sleep Apnea in Children

**DOI:** 10.21315/mjms2018.25.5.16

**Published:** 2018-10-30

**Authors:** Ramasamy Chidambaram

**Affiliations:** Department of Prosthodontics, Faculty of Dentistry, AIMST University, Jalan Bedong-Semeling, 08100 Bedong, Kedah, Malaysia

Dear Editor,

Obstructive sleep apnea (OSA) is a common serious condition that is under-recognised in children. It is characterised by multiple interruptions of breathing during sleep, which is caused by temporary obstructions of the airway. In most cases, OSA is an outcome of adenotonsillar hypertrophy, neuromuscular disease, and craniofacial abnormalities which typically peaks between ages 3–6. OSA, undoubtedly, is a major public health concern in terms of its prevalence and negative impacts (cognitive disorders, learning disabilities and emotional instability) exerted on health.

In Malaysian perspective, recent statistics suggest that 7%–9% of children have OSA, and new cases are still adding up (1). Can we say that it is due to the ignorance of parents or physicians in identifying OSA? A family medicine specialist (FMS) is the first one to detect and treat any disease(s). However, it is a pity that when a patient describes sleep-breathing disorder to FMSs, they mostly fail to link it with OSA or realise its seriousness (2). The situation becomes worse in the paediatric population as daytime sleepiness may easily be ignored by the parents and unless the parents complain loud snoring, the FMS would not be able to give a second thought to identify OSA. However, rather than arguing, we should introspect if we are missing something? In the diagnostic perspective, we have improvised a lot by formulating multiple screening tools like polysomnography and validated sleep questionnaires. Although these are well known among sleep specialists and medical experts, there is a lack of involving FMSs in the team of experts for OSA. FMSs see their patients more frequently than the specialists, especially those who adhere to recommended annual check-ups. Unfortunately, the educational backgrounds of health-care professionals create unnecessary demarcations between the expertise and FMS and speculate OSA as a specific condition.

In reality, FMSs are better off in identifying patients at a greater risk. Adenotonsillar hypertrophy and obesity are major risk factors for OSA. The adverse consequences of childhood obesity are well known and are getting more attention with the increasing prevalence of OSA, which is another double-trouble for children (3). With a brief history (snoring, mouth-breathing, bedwetting, bizarre sleeping positions, poor school performance, and aggressive behaviour) and careful observation of the conditions, considering complaints and risk factors, an FMS may recognise a possible case of OSA. FMS can employ Berlin-M (Malay version) questionnaire and earlier Malaysian studies do recommend it for primary care (4). It is sad to know that 80% of the Malaysian OSA population go undetected (5). One of the key points while handling OSA is improving general awareness and the best source to get the task done is through FMSs, because they get the opportunity to discuss the concerns that might affect their patient’s health. The Asian Paediatric Pulmonology Society also emphasise on accurate diagnosis and early recognition as the key to reduce the incidence of OSA (6). Thus, educating the FMSs about such conditions through training and educational courses can result in a better diagnosis and referral of undiagnosed OSA in children. So let’s engage the FMSs in the war on snoring and help our children to grow into successful adults.
